# A Rare Case of Iron Overload in Hereditary Spherocytosis: A Case Report

**DOI:** 10.7759/cureus.63934

**Published:** 2024-07-05

**Authors:** Audrey Bui, Avani P Shah, Min Y Chae, Peyton Popard, Bijoy Telivala

**Affiliations:** 1 Medicine, Lake Erie College of Osteopathic Medicine, Bradenton, USA; 2 Hematology and Oncology, Cancer Specialists of North Florida, Jacksonville, USA

**Keywords:** anemia, splenectomy, hemochromatosis, iron overload, hereditary spherocytosis

## Abstract

Hereditary spherocytosis (HS) is a hereditary hematologic disorder characterized by fragile spherical red blood cells that are susceptible to hemolysis. HS patients are often asymptomatic or present with anemia; however, serious complications of chronic hemolysis can include cholelithiasis and aplastic crisis. Splenectomy is considered the standard surgical treatment in moderate and severe forms of HS, with the main complication being a life-long risk of infection. Interestingly, our case suggests a possibility of secondary hemochromatosis as a complication of chronic hemolysis seen in HS. A vast majority of hemochromatosis patients possess a genetic predisposition, which increases their serum iron level and iron storage within the reticuloendothelial system. However, we present a case in which the genetic panel for common mutations associated with hemochromatosis resulted as negative. This case emphasizes the need for increased awareness regarding the potential development of idiopathic hemochromatosis in patients with long-standing HS, allowing for prompt intervention and preventing the associated complications.

## Introduction

Hereditary spherocytosis (HS) is an inherited hematological disorder characterized by spherical erythrocytes that are susceptible to hemolysis due to red blood cell (RBC) membrane defects [1]. The defects in HS result in decreased deformability and subsequent consumption of the spherocytes as they travel through the splenic sinusoids. It is the most common type of inherited anemia in those of Northern European descent, affecting approximately one in 1000 to 2500, and HS patients often are asymptomatic or present with mild-to-moderate anemia [2]. Anemia is a result of extravascular hemolysis of fragile erythrocytes and when severe, it can become poorly compensated and lead to delay in physical and sexual maturation in children. Complications of HS mainly stem from chronic hemolysis and include cholelithiasis and aplastic crises, most often in response to parvovirus B19 infection [3].

Treatment of HS focuses on improving the quality of life for patients with transfusions of packed red blood cells (pRBCs) to maintain hemoglobin (Hgb) concentration at a minimum of 7 to 8 g/dL (reference range: 12.0-16.0 g/dL) and adequate folic acid supplementation [4]. For those with severe recurrent hemolytic crises and significant splenomegaly, splenectomy is often considered to decrease extravascular hemolytic events. Studies have shown a qualitative reduction of hemolysis and transfusion rates in splenectomized patients [5]. One of the most feared complications in splenectomized HS patients is fatal sepsis due to encapsulated bacteria, such as pneumonia due to *Streptococcus pneumoniae,* and thus, additional prophylactic vaccinations and monitoring are necessary.

We propose a rare complication found in long-term HS patients: idiopathic hemochromatosis due to disruption of iron metabolism. We report a case of a 77-year-old female with HS who developed idiopathic hemochromatosis several years after splenectomy. With the increasing lifespan of HS patients, our aim is to bring awareness to this potentially rare and late complication of HS to improve patient outcomes and enhance clinical management.

## Case presentation

**Figure 1:**
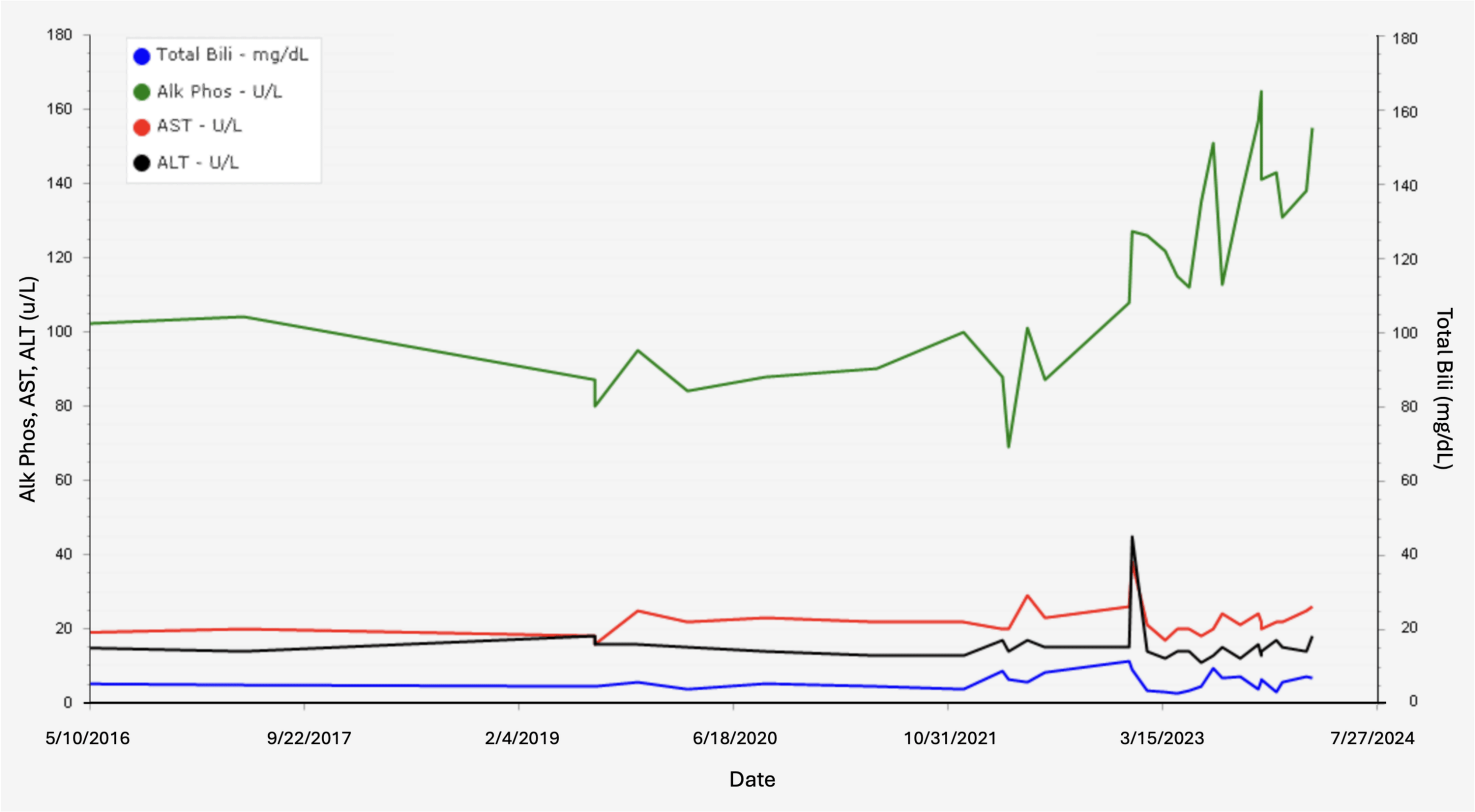
Liver panel levels from 2016 to 2024. The blue line indicates the total bilirubin level (normal is 0.2-1.2 mg/dL), the green line indicates the alkaline phosphatase level (normal is 35-104 U/L), the red line indicates the aspartate transaminase (AST) level (normal is 5-32 U/L), and the black line indicates the alanine transaminase (ALT) level (normal is 5-33 U/L).

A 77-year-old White female with a history of HS, hypertension, atrial fibrillation, and breast cancer presents with recent iron overload. The patient underwent a splenectomy several decades ago for HS with subsequent normalization of her hemoglobin levels; however, she was lost to follow-up and received intermittent care between 2005 and 2009. In 2009, her labs revealed a ferritin level of 1163 ng/mL (13.0-150.0 ng/mL) and total bilirubin of 4.3 mg/dL (0.2-1.2 mg/dL), and when she returned to the office in 2016, her bilirubin level was 5.1 mg/dL (0.2-1.2 mg/dL) (Figure 1). During this time, she had also been diagnosed with diabetes mellitus.

**Figure 2:**
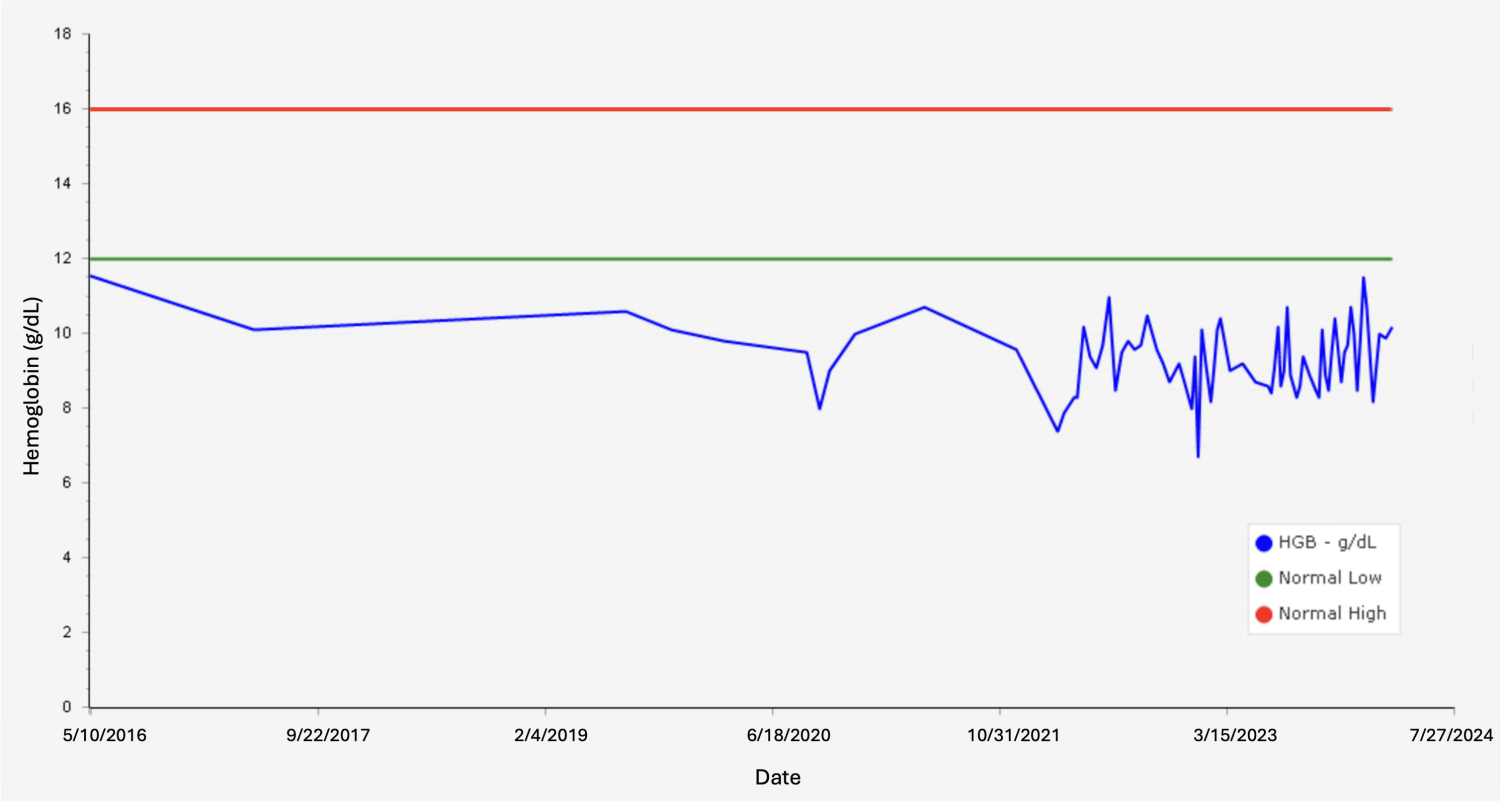
Hemoglobin (Hgb) levels from 2016 to 2024. The blue line indicates the patient's Hgb level, the green line indicates a normal low Hgb level, and the red line indicates a normal high Hgb level.

**Figure 3:**
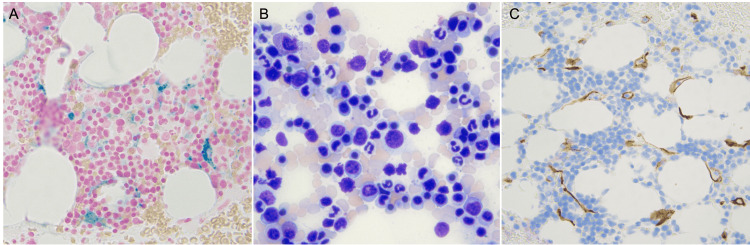
Bone marrow biopsy. (A) Histopathological examination with iron stain showed moderately increased iron stores. (B) Mild erythroid hyperplasia with orderly maturation and no dyspoiesis. (C) CD34+ blasts composed 1% (1-2%) of the bone marrow sample.

Since restarting her care, her baseline hemoglobin on average was 10 g/dL (12.0-16.0g/dL) and has lowered to 7 g/dL (12.0-16.0g/dL) since 2021 for which she received two pRBC transfusions (Figure 2). In 2022, a bone marrow biopsy was performed, which revealed increased marrow storage iron, mildly hypercellular marrow with erythroid hyperplasia, and no evidence of malignancy (Figure 3). Cytogenetic testing was also normal. Testing revealed no evidence of cold agglutinins, glucose-6-phosphate-dehydrogenase (G6PD) deficiency, or pyruvate kinase deficiency being the etiology of her anemia. Due to multiple hospitalizations that required blood transfusions and increasing concern for potential autoimmune hemolytic anemia in addition to HS, the patient was counseled on the risks and benefits of rituximab and received one 375 mg/m2 dose of rituximab per week over the course of four weeks in 2023. Her hemoglobin levels stabilized afterward, and she continued to receive erythropoietin as needed for her anemia.

**Figure 4:**
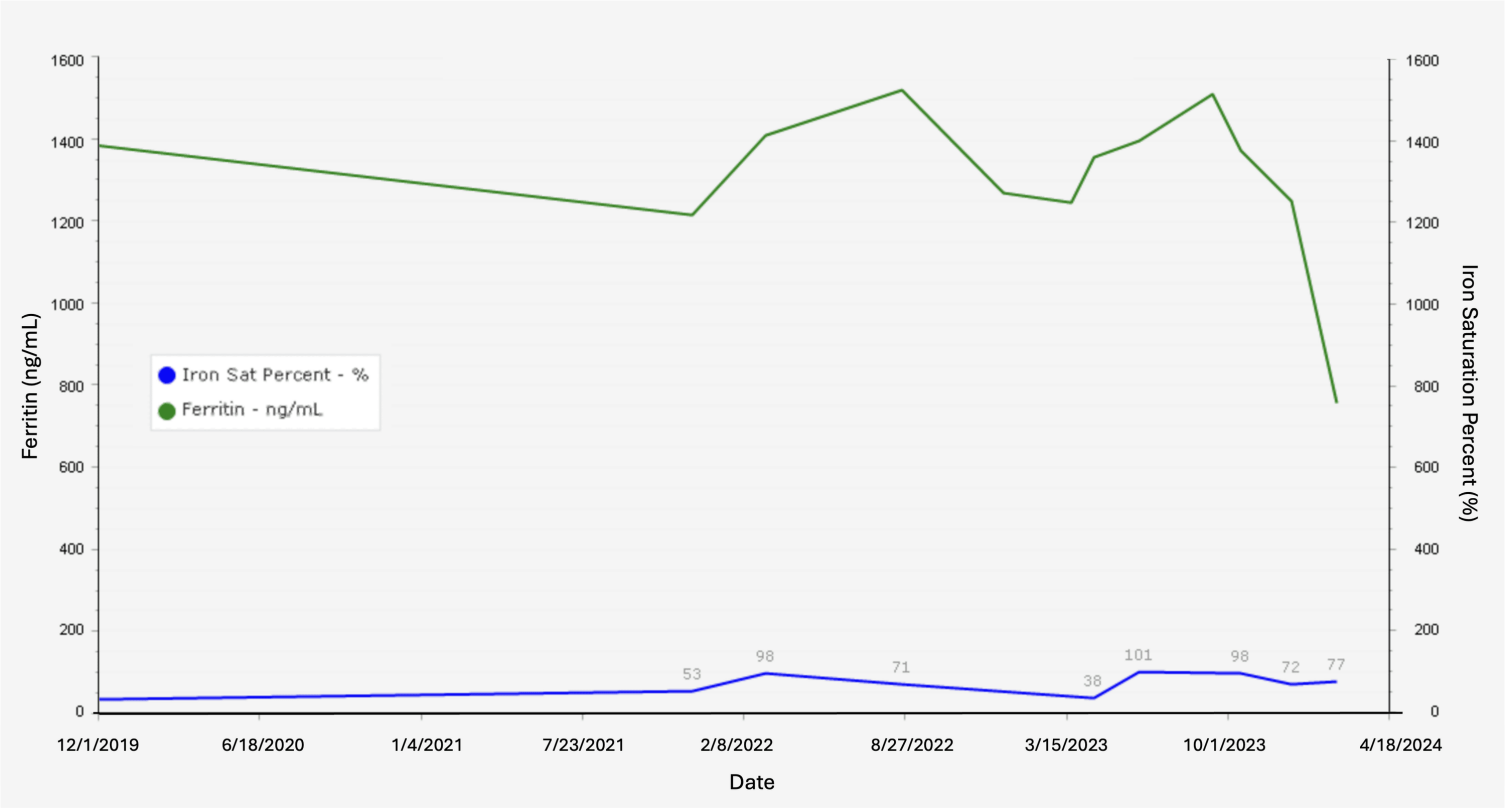
Ferritin and iron saturation percentage levels from 2019 to 2024. The blue line indicates iron saturation percent, and the green line indicates ferritin level. The normal ranges for iron saturation percentage and ferritin level are 15-55% and 13.0-150.0 ng/mL, respectively [6].

In 2023, she presented with worsening hepatomegaly and symptoms of iron overload. Her iron panel revealed ferritin at 1513 ng/mL (13.0-150.0 ng/mL) and iron saturation percentage at 98% (15-55%) (Figure 4). Liver function tests demonstrated an aspartate transaminase (AST) of 38 U/L (5-32 U/L), alanine transaminase (ALT) of 45 U/L (5-33 U/L), total bilirubin of 9.2 mg/dL (0.2-1.2 mg/dL), and alkaline phosphatase of 127 U/L (35-104 U/L) (Figure 1). A computed tomography (CT) scan was performed, which revealed moderate hepatomegaly, and a liver biopsy was subsequently performed showing evidence of iron overload. Further genetic workup for hereditary hemochromatosis remained negative, and workup for ferroportin disease was declined. Due to her anemia, she was not a candidate for phlebotomy treatment. She was started on the oral iron-chelating agent deferasirox 360 mg once per day for treatment of iron overload. Since then, her symptoms have markedly improved, and her labs have begun to normalize. At the beginning of 2024, her iron panel revealed ferritin at 756.7 ng/mL (13.0-150.0 ng/mL) and iron saturation percentage at 77% (15-55%).

## Discussion

HS is a congenital hemolytic disorder characterized by RBC membrane defects, leading to increased susceptibility to hemolysis. Splenectomy often stands as a definitive therapeutic option for managing complications of HS, such as hemolytic anemia [3]. However, this case brings to light an unexpected consequence of long-standing HS - a subsequent development of idiopathic hemochromatosis.

The observed development of hemochromatosis in our post-splenectomy patient underscores the complex interplay between erythrocyte destruction, iron metabolism, and the absence of splenic function. While the exact mechanism remains unknown, several mechanisms have been suggested to contribute to the iron overload observed in post-splenectomy patients, including chronic hemolytic anemia and subsequent increase in erythropoiesis of spherocytosis, increased intestinal iron absorption, and altered erythrocyte turnover [7]. Consequently, the unchecked accumulation of iron predisposes individuals to secondary hemochromatosis, as evident in our case [8].

Prior reports highlight the development of iron overload in long-term HS patients due to a genetic predisposition to hemochromatosis [8-12]. However, in our case, the absence of a genetic predisposition for hemochromatosis suggests that the emergence of hemochromatosis post-splenectomy could involve additional factors beyond genetic influences. This perspective underscores the need for further exploration and understanding of the intricate interactions between these conditions in different individuals.

Given the rarity of reported cases linking HS and subsequent hemochromatosis, it is imperative to recognize the potential risk factors and implement vigilant monitoring strategies in long-term HS patients even in those without a family history of hemochromatosis. Periodic assessment of iron parameters, liver function tests, and imaging studies can aid in the early detection of iron overload and prompt initiation of therapeutic interventions, thereby preventing potential complications associated with hemochromatosis such as cirrhosis, hepatocellular carcinoma, diabetes mellitus, and dilated cardiomyopathy [6].

## Conclusions

HS is a hereditary hematologic disorder characterized by fragile RBCs susceptible to hemolysis, often leading to anemia and various complications. While splenectomy remains the standard surgical intervention for moderate to severe cases, a new potential complication has been observed, i.e., secondary hemochromatosis. This finding underscores the importance of increased awareness about the possible development of idiopathic hemochromatosis in HS patients as a late complication and emphasizes the need for prompt intervention to mitigate associated risks and complications. The case serves as a valuable contribution to the evolving understanding of long-term complications in hereditary spherocytosis and calls for continued research to enhance clinical management strategies.
